# Mechanisms of the Mitochondrial Unfolded Protein Response in Caenorhabditis elegans and Mammals and Its Roles in Striated Muscles

**DOI:** 10.14336/AD.2024.1019

**Published:** 2025-11-15

**Authors:** Tongxiao Luan, Song Hu, Weihong Nie, Jia Liu, Li Jia, Shan Wang, Jing Zhou, Nina An, Yuting Duan, Aohua Wang, Mengru Xu, Yongjun Mao

**Affiliations:** ^1^Department of Health Care/Geriatrics, Affiliated Hospital of Qingdao University, Qingdao, Shandong, China.; ^2^Qingdao University, 308 Ningxia Road, Laoshan District, Qingdao, Shandong, China.; ^3^Department of emergency medicine, Affiliated Hospital of Qingdao University, Qingdao, Shandong, China.

**Keywords:** Mitochondrial unfolded protein response, UPR^mt^, Striated muscle, Cardiac muscle, Skeletal muscle, Aging

## Abstract

Throughout the course of evolution, organisms and cells have evolved a suite of mechanisms to manage persistent stimuli, thereby preserving cellular and organismal homeostasis. Upon detecting stress signals, cells activate a transcriptional response termed the mitochondrial unfolded protein response (UPR^mt^). This response is crucial for maintaining protein homeostasis, facilitating mitochondrial function recovery, promoting cell survival, and ultimately influencing lifespan. Striated muscles play a pivotal role in oxygen supply, movement, and metabolism. The aging of these muscles can lead to heart failure, arrhythmias, and sarcopenia, significantly impacting quality of life and lifespan. Given the intimate connection between UPR^mt^ and striated muscle aging, UPR^mt^ emerges as a potential therapeutic target for mitigating the effects of striated muscle aging. In this review, we delve into the role of UPR^mt^ in striated muscle aging, drawing upon the extant molecular regulatory mechanisms of UPR^mt^. This exploration may enhance our understanding of the underlying mechanisms of striated muscle aging and aid in the identification of potential drug targets.

## 1. Introduction

In the past, aging was considered an incurable disease, a physiological process that all individuals inevitably undergo on their way to death, and efforts to slow down aging were viewed as pseudoscience. Since the 1990s, with the exploration of aging mechanisms and the development of modern geriatric medicine, the possibility of slowing down aging has gradually emerged. It is predicted that in the next 30 years, the global population aged 65 and over will more than double, reaching over 1.5 billion [1]. Therefore, unless the issue of the rapidly increasing elderly population is addressed, human society will face a significant challenge in the coming decades: how to cope with the enormous burden of chronic diseases brought about by aging [2]. Unfortunately, our current understanding of the mechanisms of aging is far from sufficient.

Protein homeostasis imbalance is considered one of the key indicators of aging [3, 4]. Maintaining protein homeostasis requires a complex network to coordinate the synthesis and folding of polypeptide chains, the maintenance of protein conformation, and protein degradation. Molecular chaperone systems, protein hydrolysis systems (ubiquitin-proteasome system and autophagy-lysosome system), and regulatory factors are core mechanisms regulating protein homeostasis [5]. In fact, dealing with various internal and external stresses to maintain protein homeostasis is a challenging process, with approximately 2000 proteins involved in constructing this network [6]. Muscle fiber atrophy caused by the imbalance between muscle protein synthesis and breakdown has been proven to be an important mechanism of muscle aging [7].

Since the discovery of the mitochondrial unfolded protein response (UPR^mt^) in 1996 [8], an increasing number of scholars have found that this mechanism plays an important role in aging, leading to the publication of numerous related studies [9]. UPR^mt^ is defined as the response of regulating specific transcription factors after mitochondrial dysfunction due to various reasons (including accumulation of unfolded proteins, oxidative stress reactions, compound stimulation, and viral infections), primarily maintaining protein homeostasis, promoting mitochondrial function recovery, and enhancing cellular vitality [10, 11]. UPR^mt^ is highly conserved in organisms such as caenorhabditis elegans (C. elegans) and mammals [12]. Therefore, early models for studying the impact of UPR^mt^ on lifespan primarily focused on the C. elegans. In recent years, as the mechanisms in C. elegans have become clearer, research has shifted towards understanding the role and mechanisms of UPR^mt^ in mammals. Most studies indicate that protein homeostasis imbalance activates the UPR^mt^, maintaining protein homeostasis and playing a role in delaying aging and extending lifespan [13, 14].

**Figure 1.**
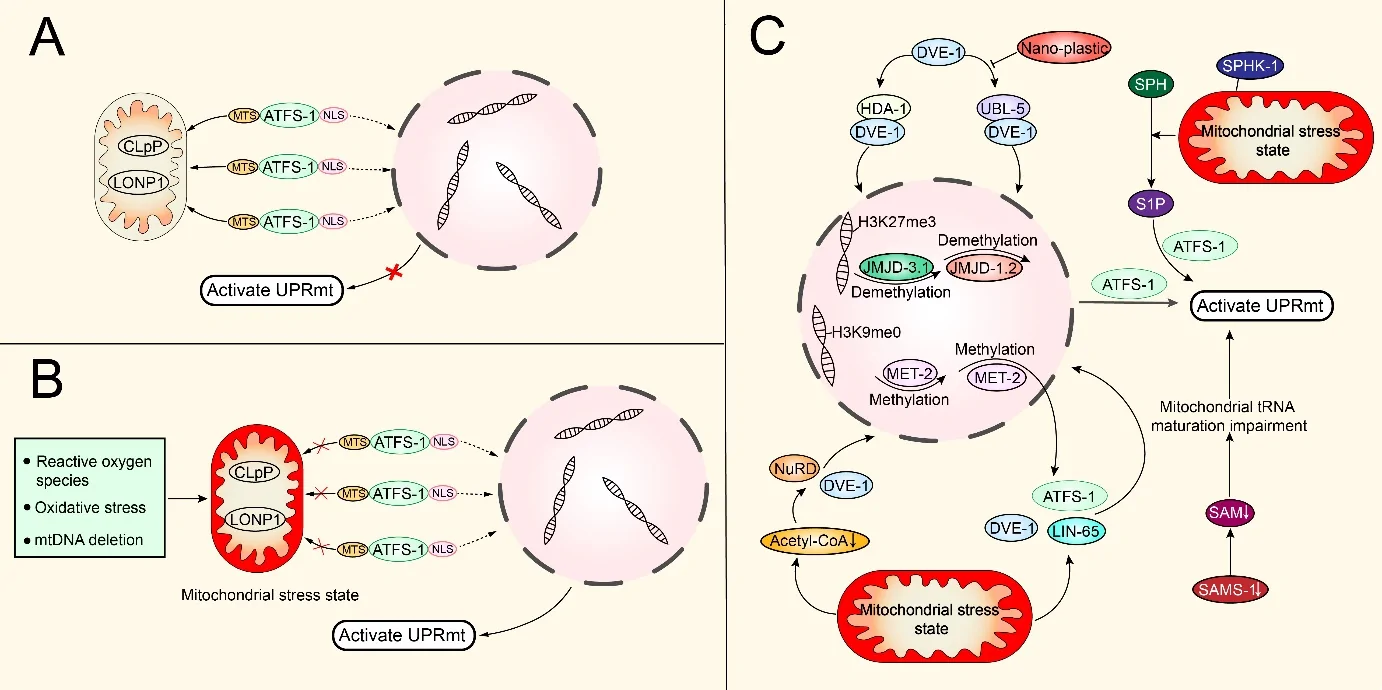
**Molecular pathways regulating autonomous mitochondrial unfolded protein response (UPR^mt^) in caenorhabditis elegans (C**. elegans). (**A**) ATFS-1 maintains a dynamic balance within the cell. Under normal conditions, ATFS-1 enters the mitochondrial matrix and is inactivated by the proteases LONP-1 and CLpP, resulting in low levels of ATFS-1 sensed by the nucleus. (**B**) Mitochondrial stress disrupts the balance of ATFS-1, activating UPR^mt^. During mitochondrial stress caused by reactive oxygen species (ROS), oxidative stress, or minor mitochondrial DNA (mtDNA) deletions, ATFS-1 fails to enter the mitochondria and thus cannot be inactivated. The nucleus senses high levels of ATFS-1, thereby activating UPR^mt^. (**C**) Regulation of UPR^mt^ in C. elegans. On one hand, DVE-1 interacts with HDA-1 to co-regulate chromatin remodeling and gene expression, activating UPR^mt^. On the other hand, DVE-1 and UBL-5 form a complex that enters the nucleus to activate UPR^mt^. Nanoplastics can inhibit the expression of UBL-5, DVE-1, ATFS-1, and CLpP-1, causing transgenerational reproductive toxicity. Under mitochondrial stress, JMJD-3.1 and JMJD-1.2 sequentially demethylate H3K27me3, activating UPR^mt^. Concurrently, histone H3K9 is continuously methylated by MET-2, leading to the accumulation of ATFS-1, DVE-1, and LIN-65 in the nucleus, activating UPR^mt^. Reduced acetyl-CoA under mitochondrial stress induces nuclear accumulation of nucleosome remodeling and histone deacetylase (NuRD) and DVE-1, decreasing histone acetylation and chromatin remodeling, thereby activating UPR^mt^. Upon mitochondrial stress activation, SPHK-1 targets the mitochondrial outer membrane and catalyzes the conversion of SPH to S1P, which transmits stress signals to activate UPR^mt^. Limiting SAMS-1 reduces mitochondrial SAM, impairing mitochondrial tRNA maturation and activating UPR^mt^. Most of those UPR^mt^ activation processes require the involvement of ATFS-1.

Striated muscle, including skeletal muscle and cardiac muscle, is essential for oxygen supply, metabolic balance, and movement [15]. Aging of cardiac muscle leads to decreased contractile force, reduced ability of cardiac muscle cells to couple with electrical pacing and is closely associated with heart failure and arrhythmias [16]. Skeletal muscle aging is a major cause of sarcopenia, leading to various risks including falls, fractures, reduced quality of life, and increased all-cause mortality [17]. The regenerative capacity of striated muscle is very low, its energy consumption is high, and the contraction of striated muscle requires the cooperation of myofilament proteins. Therefore, we hypothesize that UPR^mt^ plays a crucial role in the aging of striated muscle. To develop potential molecular targets for preventing striated muscle aging, we summarize the main molecular mechanisms of UPR^mt^ in C. elegans and mammals, and subsequently outline their roles in striated muscle aging.

## 2. Molecular Regulation of UPR^mt^

### 2.1. Cell Autonomy of UPR^mt^ in C. elegans

The activating transcription factor associated with stress-1 (ATFS-1) is the core molecule regulating cell autonomy of UPR^mt^ in C. elegans. ATFS-1 is a basic leucine zipper transcription factor that possesses both an N-terminal mitochondrial targeting sequence (MTS) and a C-terminal nuclear localization sequence (NLS) [18]. UPR^mt^ induces the transcriptional activation of 685 genes, with 391 genes requiring ATFS-1[19]. Under normal conditions, ATFS-1 can localize to both mitochondria and the nucleus, enabling it to relay mitochondrial status to the nuclear compartment. Upon its entry into the mitochondrial matrix, ATFS-1 is inactivated by lon peptidase 1 (LONP-1) [20] and caseinolytic mitochondrial matrix peptidase proteolytic subunit (CLpP) [21, 22], resulting in reduced nuclear levels of ATFS-1 (Fig. 1A). When mitochondrial stress prevents ATFS-1 from entering mitochondria, the cytoplasmic and nuclear levels of ATFS-1 increase, leading to the activation of UPR^mt^ in the nucleus [19] (Fig. 1B).This activation promotes the upregulation of glycolytic and innate immune genes, inhibits the production of tricarboxylic acid cycle and oxidative phosphorylation (OXPHOS) genes, enhances mitochondrial chaperone production, and mitigates the accumulation of intracellular reactive oxygen species (ROS) [23-25]. A moderate loss of mitochondrial DNA (mtDNA) can induce OXPHOS dysfunction, triggering ATFS-1-mediated UPR^mt^ to maintain cellular function. However, excessive activation of ATFS-1 due to severe mtDNA loss can further compromise mitochondrial function, leading to OXPHOS dysfunction [26]. Additionally, UPR^mt^ mediated by hypoxia-induced ATFS-1 activation can compensatorily improve the damage caused by hypoxia [27]. Mitochondrial stress stimulates v-ATPase/Rheb-dependent mechanistic target of rapamycin complex 1 (mTORC1) activation, promoting the translation of ATFS-1 as part of the upstream pathways [28].

Multiple molecules led by defective proventriculus in drosophila homolog 1 (DVE-1) are involved in regulating cell autonomy of UPR^mt^ in C. elegans. Early studies suggest that the accumulation of ubiquitin-like protein 5 (UBL-5) in the nucleus represents mitochondrial stress response and activates UPR^mt^ [29]. During mitochondrial stress, a complex formed by the physical interaction of UBL-5 with DVE-1 can activate UPR^mt^, regulating the transcription of genes encoding the mitochondrial heat shock protein 70 (mtHSP 70) and heat shock protein 60 (HSP 60) involved in maintaining mitochondrial homeostasis [22]. Under non-stress conditions, histone H3K9m0 is methylated by MET-2 to H3K9m1/2 in the cytoplasm, with ATFS-1, DVE-1, and LIN-65 dispersed in the cytoplasm and a relaxed nucleus where UPR^mt^ remains inactive. Upon mitochondrial stress, histone H3K9 is still methylated by MET-2, causing ATFS-1, DVE-1, and LIN-65 to aggregate in the nucleus, leading to nuclear condensation and UPR^mt^ activation [30]. It is noteworthy that UBL-5, DVE-1, MET-2, and LIN-65 enhance the activation of ATFS-1 for UPR^mt^ rather than constituting a completely new molecular pathway. Furthermore, JMJD-3.1 and JMJD-1.2 induce the expression of UBL5, HSP 60 and mtHSP 70 by sequentially demethylating H3K27me3, thereby activating UPR^mt^ [31]. Histone deacetylase (HDA-1) interacts with DVE-1 to jointly regulate chromatin remodeling and gene expression, activating UPR^mt^. Knocking out HDA-1 reduces DVE-1 expression, leading to decreased transcription levels of HSP 6 and HSP 60 and inhibiting UPR^mt^ activation [32]. During mitochondrial stress, citrate production from the tricarboxylic acid cycle decreases, followed by a decrease in acetyl-CoA levels. Acetyl-CoA serves as a signaling molecule inducing nuclear aggregation of nucleosome remodeling and histone deacetylase (NuRD) and DVE-1, reducing histone acetylation and chromatin remodeling, thereby activating UPR^mt^ and extending lifespan [33]. Interestingly, nanoplastics in the environment downregulate the transcription levels of UBL-5, DVE-1, ATFS-1, and CLpP-1, inhibiting UPR^mt^ and ultimately causing transgenerational reproductive toxicity in C. elegans [34]. Recent studies have identified ubiquitin-like protein 2 is also involved in the regulation of UPR^mt^ and mitochondrial homeostasis [35].

Sphingosine kinase 1 (SPHK-1) is widely expressed in various tissues and plays essential regulatory roles in neurotransmitter release, cell proliferation, differentiation, and apoptosis in vivo [36]. Upon activation by mitochondrial stress, SPHK-1 targets the outer mitochondrial membrane and catalyzes sphingosine conversion to sphingosine-1-phosphate (S1P). S1P acts as a second messenger molecule to transmit signals and activate UPR^mt^ [37]. Even mild activation of SPHK-1 is sufficient to significantly activate UPR^mt^ signaling, possibly due to the amplifying role of S1P in this process. It is important to note that S1P activation of UPR^mt^ still requires the presence of ATFS-1 as a foundational component. S-adenosylmethionine (SAM) is synthesized by S-adenosylmethionine synthase (SAMS) from methionine and ATP, serving as a universal methyl donor required for numerous cellular methylation reactions. In C. elegans, inhibiting SAMS-1 (the major subtype of SAMS) through gene silencing or dietary restriction significantly reduces mitochondrial SAM levels, impairing mitochondrial tRNA methylation and maturation, reducing mitochondrial translation, ultimately inducing the UPR^mt^ and extending lifespan [38]. These mechanisms are summarized in Figure 1C.

### 2.2 Cell Non- Autonomous UPR^mt^ in C. elegans

**Figure 2.**
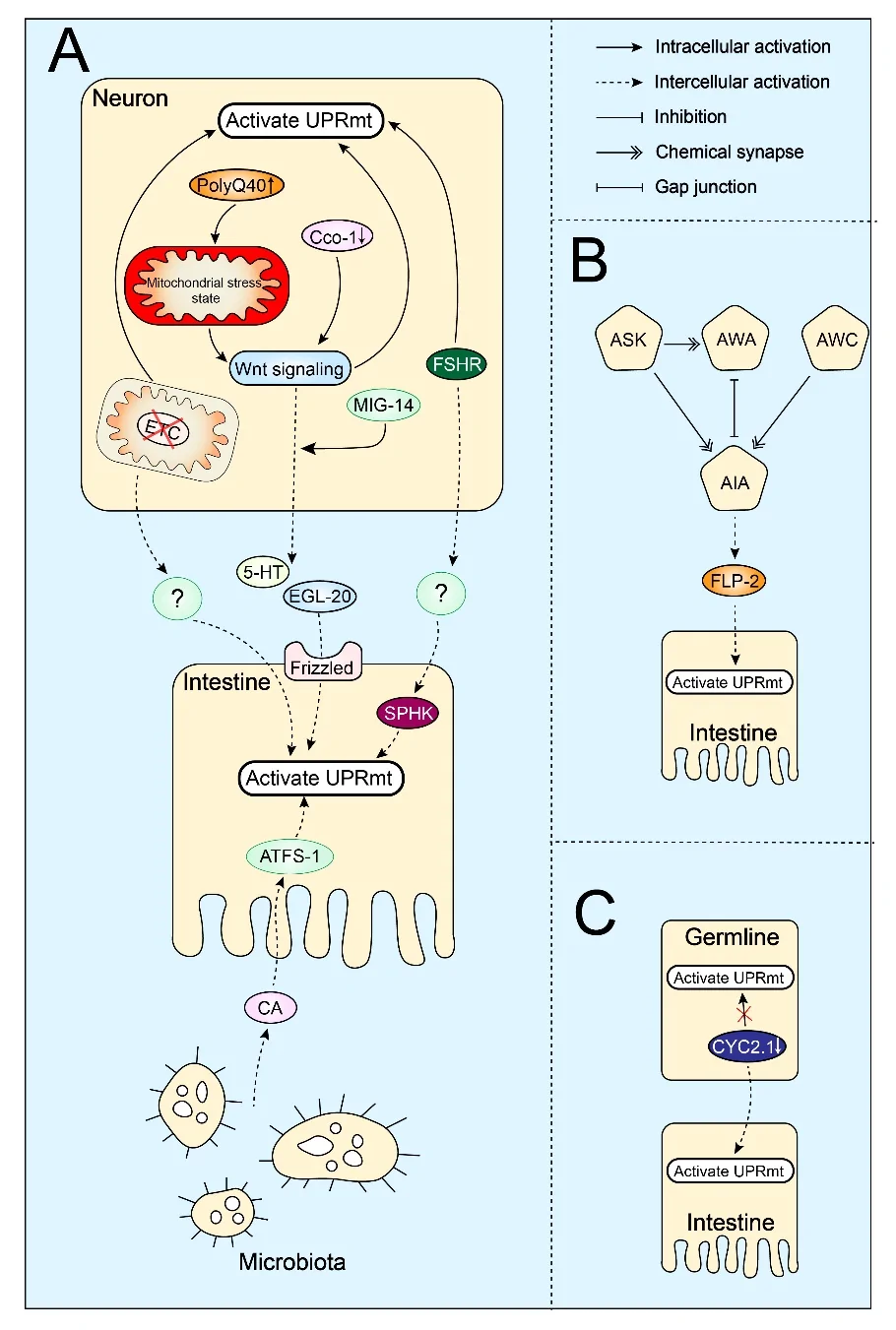
**Molecular pathways regulating cell non-autonomous mitochondrial unfolded protein response (UPR^mt^) in caenorhabditis elegans (C**. elegans). (**A**) Neurons activate cell non-autonomous UPR^mt^ in the intestine. Knocking down mitochondrial electron transport chain (ETC) complex subunits in neurons not only activates neuronal UPR^mt^ but also induces cell non-autonomous UPR^mt^ in intestine. Additionally, mitochondrial stress triggered by polyQ40 overexpression or reduced cco-1 (complex IV subunit) in neurons transmits stress signals to the intestine via the Wnt pathway with the assistance of MIG-14, activating intestinal cell non-autonomous UPR^mt^. The signaling molecules involved are serotonin and EGL-20, with Frizzled receptors in intestinal cells. Furthermore, a signaling molecule mediated by the G-protein coupled receptor FSHR-1 remotely regulates SPHK-1 in the intestine, activating cell non-autonomous UPR^mt^. The gut microbiota can also activate intestinal ATFS-1-mediated cell non-autonomous UPR^mt^ via colanic acid (CA). (**B**) A neural subcircuit composed of three types of sensory neurons (ASK, AWA, AWC) and one interneuron (AIA) activates cell non-autonomous UPR^mt^ in intestine via FLP-2. (**C**) Knockdown of cytochrome c (CYC-2.1) in germline does not induce UPR^mt^ in the germline themselves but activates cell non-autonomous UPR^mt^ in the intestine.

The molecular regulatory pathway of UPR^mt^ occurring within the same cell is termed cell-autonomous UPR^mt^. In C. elegans, UPR^mt^ can also be regulated by distant tissues [39], a phenomenon known as cell non-autonomous UPR^mt^. Early studies revealed that when the cco-1 gene (which encodes a subunit of complex IV in the mitochondrial electron transport chain, ETC) is specifically knocked out in the neurons of C. elegans, it not only activates the UPR^mt^ in the neurons themselves but also induces UPR^mt^ in intestine tissue [40, 41]. Similarly, expressing polyglutamine 40 (polyQ40) protein specifically in neurons can induce UPR^mt^ signaling in neurons while promoting the secretion of serotonin (5-HT), which then activates cell non-autonomous UPR^mt^ in the intestine [42]. Furthermore, in C. elegans neurons, polyQ40-triggered mitochondrial stress or reduction of cco-1 can, with the help of MIG-14, signal stress to the intestine via the Wnt pathway through the secretion of 5-HT and EGL-20 via the Frizzled receptor [43]. The SPHK-1 molecule in the intestine, besides being activated intracellularly, is also subject to cell non-autonomous UPR^mt^ regulation mediated by certain signaling molecules through the G-protein coupled receptor FSHR-1 in neurons [44]. Intestinal microbiota has also been shown to extend host lifespan by activating ATFS-1-mediated UPR^mt^ through polysaccharide colonic acid (CA) [12] (Fig. 2A). Further research has identified a neural subcircuit consisting of three types of sensory neurons (ASK, AWA, AWC) and an interneuron (AIA), which is essential for transmitting mitochondrial stress from neurons to the intestine. This subcircuit secretes the neuropeptide FLP-2 to convey mitochondrial stress signals from neuronal cells, thereby inducing cell non-autonomous UPR^mt^ in intestinal cells [45] (Fig. 2B). Additionally, knocking down cytochrome c (CYC-2.1) genes in germ cells of C. elegans does not induce UPR^mt^ in these cells but activates cell non-autonomous UPR^mt^ in the intestine [46] (Fig. 2C). Interestingly, multiple signaling molecules for cell non-autonomous UPR^mt^ have been identified, but the intestine remains the only known target organ. Research on cell non-autonomous UPR^mt^ is still in its early stages and requires further study as an important complement to our understanding of UPR^mt^.

### 2.3 Molecular Regulatory Pathways of UPR^mt^ in Mammals

The homologs of ATFS-1, namely transcription factor 4 (ATF-4) and activating transcription factor 5 (ATF-5), are among the core mechanisms of the mitochondrial unfolded protein response. The integrated stress response (ISR) is triggered by the phosphorylation of α subunit of eukaryotic translation initiation factor 2 (eIF2α), which can be catalyzed by four different cellular stress response kinases, to maintain or restore physiological homeostasis by activating the UPR^mt^ [24, 47, 48]. However, if the stress persists and homeostasis cannot be maintained, the ISR will trigger apoptosis to eliminate damaged cells [49]. These four kinases respond to different stimuli: general control nonderepressible 2 (GCN2) is typically activated by amino acid depletion, PKR-like endoplasmic reticulum kinase (PERK) is activated by endoplasmic reticulum stress, protein kinase R (PKR) is activated by viral infection, and heme-regulated inhibitor (HRI) is activated by heme deficiency [50-54]. Phosphorylation of eIF2α by these kinases [55] inhibits protein translation within the cell, thereby reducing the folding load on chaperone proteins [49, 56]. Conversely, transcription factors activating ATF-4, ATF-5, and C/EBP homologous protein (CHOP) are preferentially translated, inducing the expression of UPR^mt^-related genes to correct misfolded proteins [57, 58]. DELE1 appears to play a crucial role in this process. Stress-induced activation of OMA1 leads to DELE1 cleavage and accumulation in the cytoplasm, where it binds and activates HRI through its C-terminal portion, ultimately assisting HRI in phosphorylating eIF2α [47]. Furthermore, DELE1 can assist the translation of ATF4 to circumvent global translation suppression caused by eIF2α phosphorylation, thereby promoting the translation of ATF4 [48].

CHOP, ATF4, and ATF5 are considered key transcription factors for inducing UPR^mt^ in mammals [59, 60]. However, their coordination during mitochondrial dysfunction is not fully understood. All three proteins are basic leucine zipper proteins and homologous to ATFS-1 [61]. Unlike CHOP and ATF4, ATF5 contains an MTS that can rescue UPR^mt^ activation in ATFS-1-deficient C. elegans [59]. While ISR and UPR^mt^ molecular regulation are considered equivalent, they have some differences [10, 62]. In ISR, ATF4 acts upstream of ATF5 and CHOP [63]. During UPR^mt^, however, CHOP, ATF4, and ATF5 are independently activated, although the absence of CHOP and ATF4 reduces ATF5 transcription and expression [64], suggesting that ATF5 transcription depends on CHOP and ATF4 [10, 51, 65, 66]. However, studies have also indicated that knocking out ATF4 and CHOP, the main effectors of ISR, does not significantly affect UPR^mt^ activation [67]. Knocking out peroxisome proliferator-activated receptor gamma coactivator 1-alpha (PGC1-α) reduces ATF5 activation of UPR^mt^, indicating that PGC1-α may be an upstream molecule for ATF5 [68]. While CLpP plays a central role in regulating UPR^mt^ in C. elegans, it is neither essential nor involved in regulating mammalian UPR^mt^ [69]. Stimulation of mitochondrial stress by v-ATPase-mediated lysosomal pathways can activate mTORC1 signaling target point, which then directly phosphorylates mitochondrial stress response transcription factor and activates ATF4 [70].

The sirtuin family is involved in regulating UPR^mt^ in mammals. The sirtuin family consists of seven proteins with deacetylase activity targeting both histones and non-histone proteins, regulating metabolism, stress responses, and aging [71]. Increasing nicotinamide adenine dinucleotide (NAD(+)) levels in mice can upregulate SIRT1 and ultimately activate UPR^mt^. This process not only reduces the mRNA encoding CDKN1A, activates the expression of OXPHOS-related proteins, delays the aging of mouse muscle stem cells, neural stem cells, and melanocyte stem cells [72], but also upregulates the expression of HSP 60 and CLpP proteins, extending the lifespan of mice [73]. SIRT3 is suggested to bridge antioxidant mechanisms, mitophagy, and UPR^mt^ [74-76]. The SIRT3-AMPK pathway activates UPR^mt^ to inhibit cardiac hypertrophy and alleviate cardiac dysfunction [77] independently of CHOP and estrogen receptor α (ERα) pathways [78]. During the aging process, the expression of SIRT7 in hematopoietic stem cells decreases. However, high expression of SIRT7 inhibits mitochondrial protein folding stress and compensatory activation of UPR^mt^ by interacting with nuclear respiratory factor 1 (NRF1), thereby improving the energy metabolism and regenerative capacity of hematopoietic stem cells [79].

Mitochondrial stress response, mtDNA deletions and mutations are important molecular mechanisms regulating UPR^mt^. During mitochondrial intermembrane space (IMS) stress responses, phosphorylation of protein kinase B (AKT) and overexpression of ROS trigger ERα activity. Then ERα upregulates NRF1 and High-temperature requirement A2 (HtrA2) to activate UPR^mt^ [80]. Besides activating the ERα-NRF1-HtrA2 pathway, IMS stress responses can independently induce Sirt3-FOXO3a pathway and CHOP protein expression, jointly activating UPR^mt^ [81]. When the expression of ERα is inhibited, the CHOP protein pathway can play a compensatory role in activating UPR^mt^ to maintain mitochondrial function [82]. Additionally, due to differences in ERα expression in different genders, the UPR^mt^ induced by the female ERα-NRF1- HtrA2 is stronger than that in males [82].

Under physiological conditions, single-stranded DNA-binding protein 1 (SSBP1) predominantly exists in the mitochondrial matrix as a tetramer binding to single-stranded mtDNA, ensuring smooth mitochondrial replication and mtDNA stability [83, 84]. During mitochondrial stress, SSBP1 exits mitochondria via the ANT-VDAC1 complex to form a complex with heat shock transcription factor 1 (HSF1) that translocates to the nucleus to upregulate HSP and activate UPR^mt^ [84]. Interestingly, overexpression of mitochondria-targeted HSF1 inhibits the formation of SSBP1 oligomers, ultimately leading to mtDNA depletion [85]. Mitochondrial misfolding stress can lead to defects in mitochondrial protein import and the accumulation of mitochondrial protein precursors in the cytosol (c-mtProt), which subsequently results in the release of mitochondrial reactive oxygen species (mtROS) into the cytoplasm. Released mtROS oxidize DNAJA1(a member of HSP 40), enhancing cytosolic HSP 70 recruitments to c-mtProt, releasing HSF1 to translocate to the nucleus and activate UPR^mt^ gene transcription [67].

Reduced matching degree between mtDNA and nuclear DNA, and imbalance of encoded proteins due to mtDNA mutations activate UPR^mt^ to improve lifespan—a common pathway for longevity compounds like resveratrol and rapamycin targeting different molecular targets [86, 87]. In mouse intestines, increased mtDNA mutations activate UPR^mt^ via ATF5 and this aging phenotype can be reversed by supplementing with nicotinamide mononucleotide (NMN) [88].

**Table 1:** Related signal molecules of the mitochondrial unfolded protein response in Caenorhabditis elegans and mammals.

Core molecules	Related molecules	Reference.
**C. elegans**		
ATFS-1	ATFS-1	19, 23-25
	LONP-1, CLPP	20-22
	mtDNA	26
	Hypoxia	27
	mTORC1	28
	UBL-5, DVE-1, MET-2, LIN-65	22, 29, 30
	JMJD-3.1, JMJD-1.2	31
	HDA-1	32
	Mitochondrial stress	33
	ULP-2	35
	S1P, SPHK	37
**SAMS1-SAM**		38, 44
**Mammals**		
ATF5	eIF2α	49, 55, 56
	**ATF4, ATF5, CHOP**	10, 51, 57-60, 64-68, 70
	**DELE1**	47, 48
	**CLPP**	69
Sirtuin family	SIRT1	72, 73
	**SIRT3**	74-78, 81
	**SIRT7**	79
**ERα-NRF1-HtrA2**		80, 82
**HSF1**	mtDNA, SSBP1, mtROS	67, 84, 85
**mtDNA**	Resveratrol, Rapamycin, ATF5, NMN	86-90

Note: C. elegans, caenorhabditis elegans; ATFS-1, activating transcription factor associated with stress-1; LONP-1, lon peptidase 1; CLpP, caseinolytic mitochondrial matrix peptidase proteolytic subunit; mtDNA, mitochondrial DNA; mTORC1, mechanistic target of rapamycin complex 1; UBL-5, ubiquitin-like protein 5; DVE-1, defective proventriculus in drosophila homolog 1; HDA-1, histone deacetylase; ULP2, ubiquitin-like protein 2; S1P, sphingosine-1-phosphate; SPHK-1, Sphingosine kinase 1; SAMS, S-adenosylmethionine synthase; SAM, S-adenosylmethionine; ATF5, activating transcription factor 5; eIF2α, α subunit of eukaryotic translation initiation factor 2;ATF4, activating transcription factor 4; CHOP, C/EBP homologous protein; ERα, estrogen receptor α; NRF1, nuclear respiratory factor 1; HtrA2, High-temperature requirement A2; HSF1, heat shock transcription factor 1; SSBP1, single-stranded DNA-binding protein 1; mtROS, mitochondrial reactive oxygen species; NMN, nicotinamide mononucleotide.

**Figure 3.**
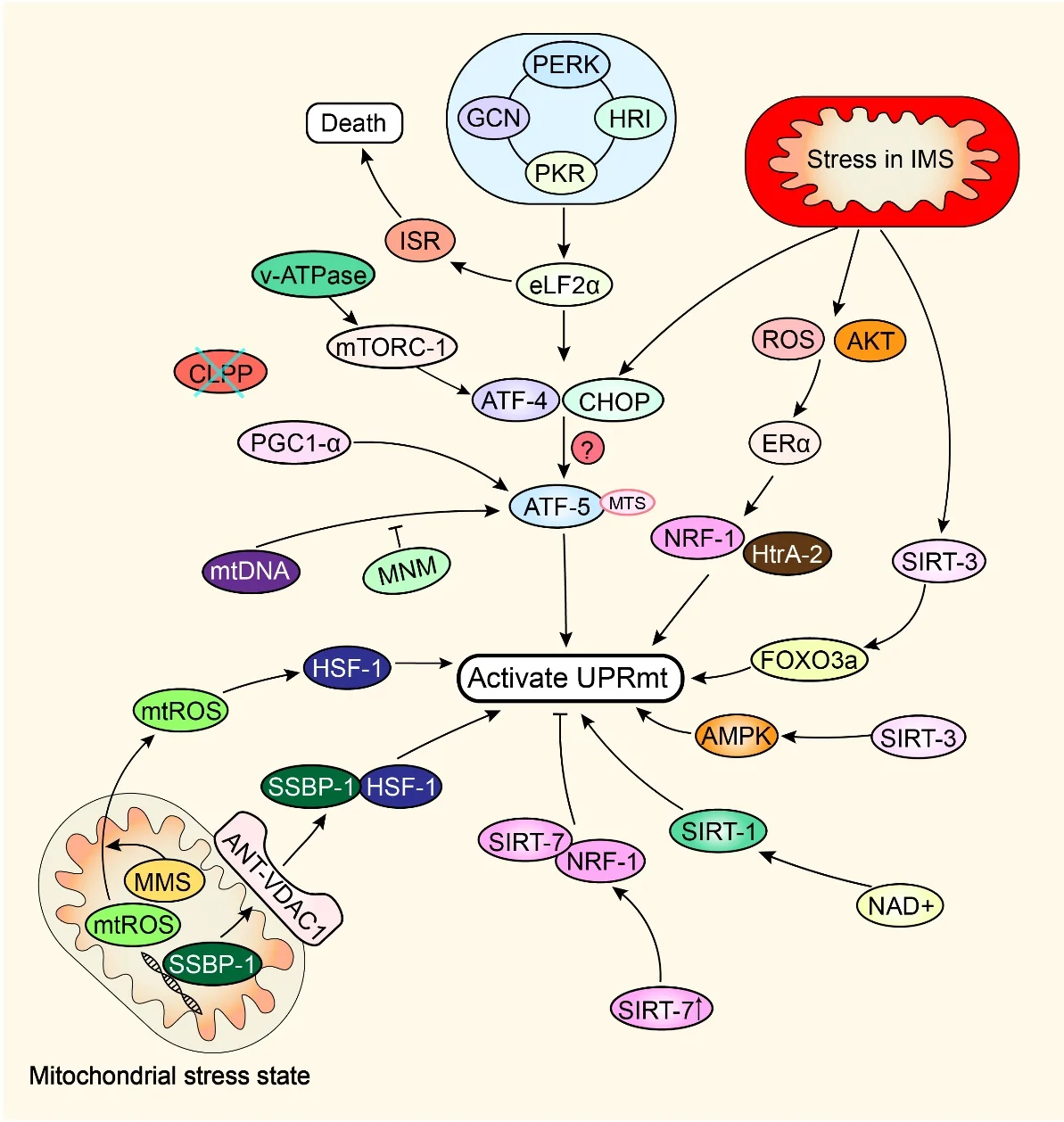
**Molecular Pathways Regulating mitochondrial unfolded protein response (UPR^mt^) in Mammals**. EIF2α is phosphorylated by four kinases: GCN2, PERK, PKR, and HRI, leading to the preferential translation of transcription factors ATF4, CHOP, and ATF5, thereby activating UPR^mt^. This process may involve upregulation of CHOP and ATF4, which subsequently induce ATF5 expression. Excessive activation of the ISR can lead to cell death via apoptosis, pyroptosis, and ferroptosis. During stress in the mitochondrial intermembrane space (IMS), phosphorylation of AKT and overexpression of reactive oxygen species (ROS) trigger ERα activity, further upregulating the transcription of HTRA2 and mitochondrial regulator NRF1, activating UPR^mt^. Concurrently, CHOP protein expression and the Sirt3-FOXO3a pathway are independently induced, jointly activating UPR^mt^. The SIRT3-AMPK pathway also activates UPR^mt^. Increasing NAD+ levels upregulate SIRT1, activating UPR^mt^. High expression of SIRT7 inhibits compensatory activation of UPR^mt^ through interaction with NRF1. Under mitochondrial stress, SSBP1 is expelled from the mitochondria via the ANT-VDAC1 complex, forming a complex with HSF1, which translocates to the nucleus to activate UPR^mt^. Mitochondrial misfolded stress (MMS) releases mitochondrial reactive oxygen species (mtROS) into the cytoplasm, causing HSP70 to release HSF1, which then translocates to the nucleus to activate UPR^mt^. Increased mtDNA mutations activate UPR^mt^ via the ATF5 pathway, and supplementation with nicotinamide mononucleotide (NMN) can reverse UPR^mt^ activation. PGC1-α may be an upstream molecule of ATF5. CLpP is neither essential for UPR^mt^ nor involved in its regulation. Stimulation of mitochondrial stress by v-ATPase-mediated lysosomal pathways can activate mTORC1 signaling target point, which then directly activates ATF4

Human mitochondrial myopathies with mtDNA replication defects also activate UPR^mt^ [89]. Patients with multiple mtDNA deletions show increased local UPR^mt^ signaling molecules around respiratory chain-deficient perinuclear foci containing mtDNA deletions [90]. Interestingly, since UPR^mt^ can sustain and propagate harmful mtDNA, long-term activation of UPR^mt^ could potentially be detrimental [26]. These mechanisms are summarized in Figure 3. Related signal molecules of the mitochondrial unfolded protein response in C. elegans and mammals are summarized in Table 1.

## 3. Myocardial Aging and UPR^mt^

Cardiomyocytes constitute approximately 30% to 35% of the total number of heart cells and account for 80% of the total volume of heart [91], making them crucial for the heart's ejection function. From age 1 to 20, human cardiomyocytes can proliferate rapidly, increasing in number by approximately 3.4 times [92]. Throughout the rest of life, the number of these cells does not significantly change, with less than 40% of cardiomyocytes undergoing renewal at a rate of about 0% to 4% per year [92-94]. Therefore, in adulthood, adaptive hypertrophy of cardiomyocytes is the primary method by which the body regulates heart function. [94] With aging, the incidence of pathological myocardial hypertrophy significantly increases due to a combination of various factors, posing a severe threat to the health of the elderly [95]. Pathological myocardial hypertrophy leads to an inability of the ventricles to relax effectively, resulting in insufficient ventricular filling and reduced end-diastolic volume, but does not impair the patient's ejection fraction. This is an important pathogenesis mechanism for heart failure with preserved ejection fraction (HFpEF) that appears during the aging process [96]. Therefore, reversing myocardial hypertrophy and improving cardiovascular health are the focus of research on delaying cardiac aging in recent years.

### 3.1 UPR^mt^ acts as a compensatory protective mechanism in cardiac aging

Inhibiting UPR^mt^ activation by silencing the type II iodothyronine deiodinase leads to severe impairment of mitochondrial respiration and extensive mitochondrial fragmentation [97]. Conversely, upregulating ATF5 to activate UPR^mt^ has a significant protective effect on the heart, suggesting that UPR^mt^ activation might be a potential therapeutic target for heart protection [98]. Short-term use of cocaine induces UPR^mt^ activation to compensate for its toxicity to cardiomyocytes, a mechanism that depends on increased expression and nuclear translocation of ATF5 [99]. Additionally, the abundance of UPR^mt^-related proteins significantly increases in heart failure animal models [100]. Similarly, the mRNA expression levels of UPR^mt^ marker genes such as HSP 70, HSP 75, and HSP 10 are markedly higher in the hearts of p32-deficient mice, indicating that UPR^mt^ is also activated in p32-deficient hearts [101]. This suggests that UPR^mt^ can act as a compensatory response to protect cardiac homeostasis when myocardial cell homeostasis is disrupted.

Interestingly, in C. elegans, disruption of the ETC activates UPR^mt^ and extends lifespan [102]. In mammals, using complex III inhibitors to separately inhibit mature cardiomyocytes HL-1 and immature cardiomyoblasts H9C2 reveals that HL-1 cells exhibit significant cell cycle arrest and death, while H9C2 cells only show mild cell death without affecting cell proliferation [103]. Early studies in C. elegans also demonstrate that UPR^mt^ activation only extends lifespan during development, but not in adulthood, where it can even have adverse effects [41]. These results suggest that UPR^mt^ activation leads to different outcomes depending on the age and type of the cells.

### 3.2 Activation of UPR^mt^ can improve cardiac remodeling and hypertrophy

Overexpression of C1q-tumor necrosis factor-related protein-3 (CTRP3) in hypertrophic cardiomyopathy mice can activate UPR^mt^ through the SIRT1-ATF5 pathway, thereby alleviating myocardial hypertrophy [104]. In both in vivo and in vitro experiments with rats, choline was demonstrated to activate UPR^mt^ through the SIRT3-AMPK signaling pathway, thus improving myocardial remodeling and hypertrophy [77]. Tetrahydrocurcumin was shown to improve myocardial remodeling and hypertrophy in rats by regulating UPR^mt^ activation via the PGC-1α/ATF5 axis [68]. These findings suggest that multiple molecules and pathways may be involved in the molecular regulation of myocardial hypertrophy by UPR^mt^.

Monogenic mutations and hypertension are significant causes of pathological hypertrophic cardiomyopathy [105]. Monogenic variations account for approximately 30% to 60% of hypertrophic cardiomyopathy cases and are primarily caused by mutations in genes encoding sarcomere proteins [106]. Analysis of heart samples from patients with sarcomere protein gene mutations positive for hypertrophic cardiomyopathy revealed significantly higher levels of HSP compared to those negative for sarcomere protein gene mutations, possibly due to the activation of UPR^mt^ by these mutations [107]. Hypertension-induced hypertrophic cardiomyopathy is characterized by sympathetic nervous excitation and cardiac mitochondrial abnormalities [108]. Sympathetic nervous excitation can inhibit miR-18a-5p/HIF-1α signaling, increase mitochondrial stress protein toxicity, reduce UPR^mt^ activation, and ultimately induce myocardial hypertrophy [109]. UPR^mt^ may play a key role in myocardial hypertrophy and remodeling.

### 3.3 UPR^mt^, autophagy, and apoptosis in cardiomyocytes appear to be cellular choices under different mitochondrial stress intensities

Brg1/Brm double mutant mice show a decrease in mitochondrial numbers, and besides the upregulation of UPR^mt^-related proteins, mitochondrial autophagy also increases [110]. Mild mitochondrial stress activates both UPR^mt^ and mitochondrial autophagy, hindering age-dependent deterioration of skeletal muscle function and structure, and extending the lifespan of Drosophila [102]. Quercetin has also been shown to protect myocardial tissue by regulating the mitophagy-UPR^mt^ pathway [104]. These results indicate that mitochondrial autophagy and UPR^mt^ are somewhat interrelated, and both work synergistically to maintain mitochondrial metabolism, respond to mitochondrial oxidative stress, and enhance cardiomyocyte vitality [111, 112]. Interestingly, activating mitophagy in cardiomyocytes does not significantly affect UPR^mt^ activation, whereas inhibiting mitophagy enhances UPR^mt^. Additionally, inhibiting UPR^mt^ reduces the protective effects of mitochondrial autophagy and UPR^mt^ on the heart, suggesting that UPR^mt^ might serve as a compensatory mechanism when mitophagy is inhibited [113].

Mutant ornithine transcarbamylase(ΔOTC) trigger UPR^mt^ to clear ΔOTC and restore protein homeostasis. Knocking down NRF1 to inhibit UPR^mt^ results in the accumulation of ΔOTC and induces apoptosis [79]. In cardiomyocytes, calreticulin may activate UPR^mt^ through the regulation of proteins such as ATF4 and CHOP, thereby protecting cardiomyocytes from angiotensin II-induced apoptosis [114]. These results suggest that UPR^mt^ can clear misfolded proteins and reduce the occurrence of apoptosis.

In fact, the relationship between UPR^mt^, mitochondrial autophagy, and apoptosis is quite complex. Some drugs or molecules can simultaneously activate them. For example, simvastatin can concurrently activate UPR^mt^, autophagy, and apoptosis, collectively regulating the homeostasis of human atrial fibroblasts [115]. Previous studies have shown that apigenin can improve mitochondrial function in cardiomyocytes through autophagy and apoptosis [116, 117]. Recent research further demonstrates that apigenin increases cardiac transcription of UPR^mt^-related genes, reduces myocardial swelling, inhibits cardiac inflammation, and significantly restores doxorubicin-induced cardiotoxicity. This protective effect appears to be related to the activation of SIRT1-ATF5, as blocking UPR^mt^ activation or knocking down SIRT1 in vitro eliminates the protective effects of apigenin on cardiomyocytes [118]. Moreover, upregulating the anti-apoptotic molecule Bax inhibitor-1 (BI-1) can improve myocardial injury in type 3 cardiorenal syndrome patients by activating UPR^mt^ and FUNDC1-related mitophagy [119]. Selectively targeting small amounts of HSP 90 to the mitochondria of human tumor cells can trigger compensatory UPR^mt^ and mitophagy to maintain cellular homeostasis; whereas large amounts of HSP 90 suppress tumor growth and promote tumor apoptosis [120]. In contrast, palmitic acid dose-dependently increases the expression of UPR^mt^ markers (HSP 60, LONP1, ATF4, and ATF5), leading to elevated levels of cytochrome c, cleaved caspase-3, and Bax/Bcl2, ultimately resulting in apoptosis. Baicalein reverses UPR^mt^ activation through the NRF2 signaling pathway, inhibiting mitochondrial-mediated apoptosis and providing protective effects on cardiomyocytes [121]. So far, most studies suggest that activating UPR^mt^ helps maintain cardiomyocyte homeostasis and function, although at different stages [103] or excessive activation [122] of UPR^mt^ may impair cardiomyocytes. The relationship between UPR^mt^, mitochondrial autophagy, and apoptosis is very close, and the three seem to represent different choices made by cells under varying intensities of mitochondrial stress. A simple hypothesis is that low-intensity stress activates UPR^mt^ to restore mitochondrial and cellular homeostasis. As stress intensity increases, mitochondrial autophagy is activated to clear proteins and mitochondria that UPR^mt^ cannot restore, maintaining cellular homeostasis. When stress intensity becomes even higher, apoptosis is initiated to eliminate cells that cannot maintain homeostasis, thus protecting other cells. Logically, this hierarchical approach allows for more efficient handling of different levels of cellular stress. Curiously, the ubiquitin-proteasome system is involved in UPR^mt^ activation in C. elegans [123, 124], Drosophila [125], and humans [126, 127]. However, so far, there seems to be no evidence of UPS involvement in UPR^mt^ activation in mammalian hearts.

## 4. Skeletal Muscle and UPR^mt^

Skeletal muscle accounts for more than 40% of body weight. Its aging mechanisms primarily include the disruption of homeostasis in protein synthesis and degradation in terminally differentiated muscle fibers, and a decline in the activity of elderly muscle stem cells leading to regeneration defects [128]. Skeletal muscle aging has long been considered a powerful driver of overall body aging. There are two types of muscle fibers in skeletal muscle: fast-twitch muscle fibers, which react quickly and have high explosive power, and slow-twitch muscle fibers, which react slowly and have high endurance. With age, fast-twitch muscle fibers deteriorate rapidly, and some of them gradually transition into slow-twitch muscle fibers [129]. The most direct consequence of skeletal muscle aging is the occurrence of sarcopenia. Sarcopenia is defined as an age-related loss of skeletal muscle mass combined with a loss of muscle strength and/or reduced physical performance [130]. Sarcopenia can lead to rapid decline in muscle mass and function, associated with increased risks of falls, functional decline, weakness, and mortality [131], making it an increasingly serious public health issue for the elderly. Clinically, severe sarcopenia has been shown to be closely associated with catastrophic healthcare expenditures [132]. Exercise, especially resistance training, is recognized as a beneficial way to improve sarcopenia [133, 134].

### 4.1 UPR^mt^ regulates skeletal muscle aging

Activating UPR^mt^ can improve mitochondrial function in skeletal muscle and delay its aging [135]. On one hand, acipimox, a precursor substance of NAD(+), improves mitochondrial function in human skeletal muscle by activating UPR^mt^ [136]. Additionally, silencing miR-382-5p can induce mitonuclear protein imbalance and activate UPR^mt^ in skeletal muscle, thereby improving mitochondrial metabolism [137]. In Drosophila, mitochondrial stress in muscles not only activates UPR^mt^ but also triggers upregulation of ImpL2, ultimately extending lifespan [102]. Conversely, knocking out HtrA2/Omi (a protein regulating IMS protein homeostasis) in vitro leads to IMS protein imbalance, activating UPR^mt^ and ultimately inducing sarcopenia [138]. However, knocking out HtrA2/Omi gene in mice does not activate UPR^mt^ but rather induces protein imbalance and ROS bursts, leading to mitochondrial dysfunction [139]. On the other hand, the NAD(+) precursor nicotinamide riboside (NR) induces UPR^mt^ and prohibits proteins synthesis, restoring muscle stem cells vitality in aged mice and preventing muscle stem cells senescence in the mdx mouse model of muscular dystrophy [72]. Furthermore, the combination of LIN28A, TERT, and sh-p53 activates UPR^mt^ through protein damage, ultimately delaying the senescence of muscle progenitor cells [140]. Additionally, decreased Ca2+ abundance leading to increased Ca2+-dependent proteolysis and reduced protein translation, ultimately inhibiting UPR^mt^, might be one of the mechanisms underlying sporadic inclusion body myositis [141]. Severe burns increase protein damage in patients and activate UPR^mt^, upregulating mitochondrial-specific proteases (LONP1 and CLpP) and mitochondrial translocases (TIM23, TIM17B, and TOM40) to cope with the hypermetabolic and hypercatabolic state induced by burns in skeletal muscles [142].

During aging and heat stress treatment, activation of UPR^mt^ in soleus muscle tissue was most evident compared to gastrocnemius and plantaris muscles, effectively improving mitochondrial stress and maintaining mitochondrial health. This might be because soleus muscle is an oxidative metabolism-dominant skeletal muscle with higher mitochondrial content [143]. The loss of cannabinoid receptor 1 can simultaneously induce the transition from fast-twitch muscle fibers to slow-twitch muscle fibers and enhance UPR^mt^ [144]. After high fat diet (HFD) intervention, the expression levels of UPR^mt^ marker proteins like ATF5, HSP 60, and ClpP were significantly higher in slow-twitch muscle fibers compared to fast-twitch muscle fibers, suggesting that UPR^mt^ might be involved in the resistance of slow-twitch muscle fibers to HFD [145].

### 4.2 Activation of UPR^mt^ is one of the key mechanisms through which exercise impacts the body

**Figure 4.**
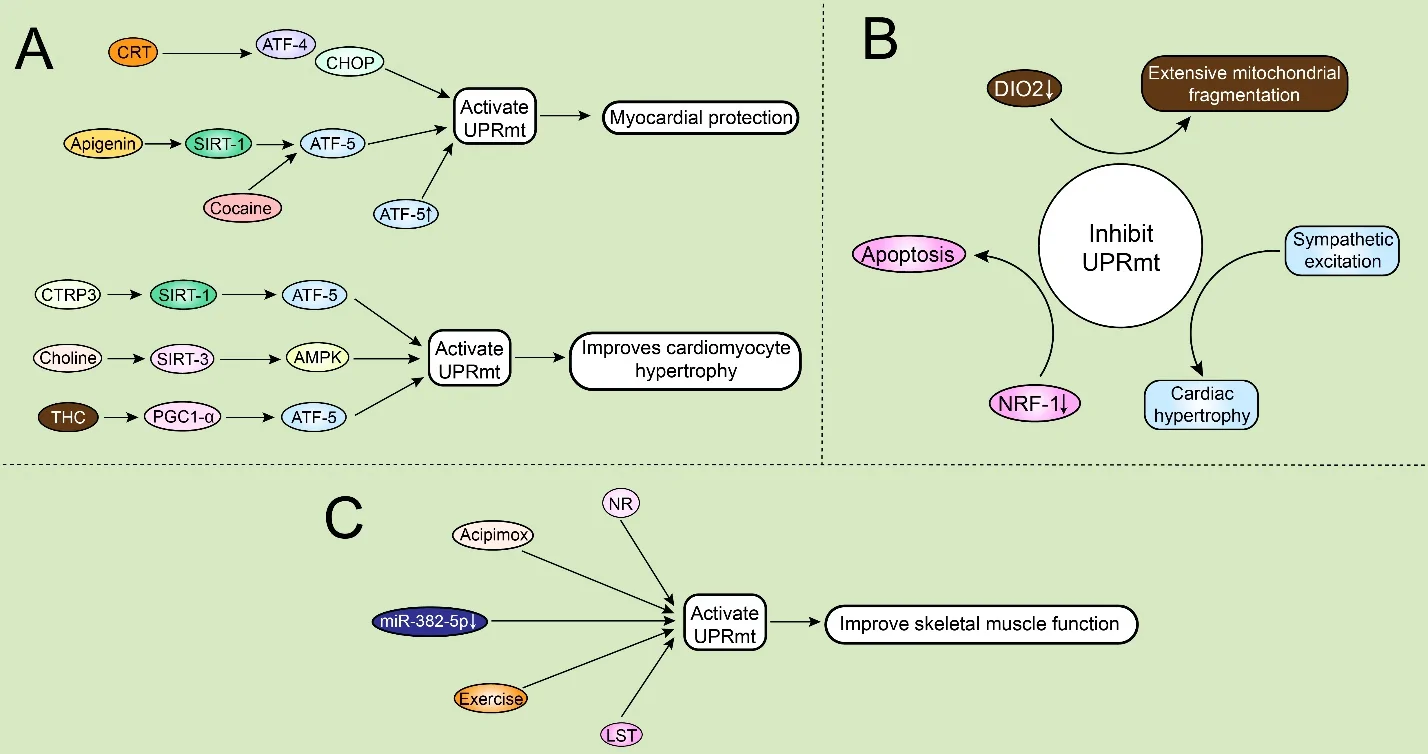
**Function of mitochondrial unfolded protein response (UPR^mt^) in myocardium and skeletal muscle**. (**A**) Activation of UPR^mt^ can exert cardioprotective effects and delay cardiac remodeling. Calreticulin (CRT) may activate mitochondrial unfolded protein response (UPR^mt^) by regulating proteins such as ATF4 and CHOP, reducing cardiomyocyte apoptosis. Apigenin activates UPR^mt^ via the SIRT1-ATF5 pathway, reducing myocardial swelling, inhibiting cardiac inflammation, and restoring cardiotoxicity caused by doxorubicin. Short-term cocaine use induces the expression of ATF5, activating UPR^mt^ to compensate for its toxicity to cardiomyocytes. Upregulating ATF5 to activate UPR^mt^ has a significant protective effect on the heart. Furthermore, overexpression of C1q-tumor necrosis factor-related protein-3 (CTRP3) can activate UPR^mt^ via the SIRT1-ATF5 pathway, thereby alleviating cardiac hypertrophy. Choline can improve cardiomyocyte remodeling and hypertrophy by activating UPR^mt^ through the SIRT3-AMPK pathway. Tetrahydrocurcumin (THC) improves cardiomyocyte remodeling and hypertrophy by activating UPR^mt^ through regulating the PGC-1α-ATF5 pathway. (**B**) Inhibition of UPR^mt^ can lead to myocardial damage. Silencing type II iodothyronine deiodinase (DIO2) to limit the activation of UPR^mt^ leads to extensive mitochondrial fragmentation in myocardium. Sympathetic excitation can reduce the activation of UPR^mt^, and ultimately induce cardiac hypertrophy. Knockdown of NRF1 to suppress UPR^mt^ induces apoptosis. (**C**) Activation of UPR^mt^ can improve skeletal muscle function. Nicotinamide riboside (NR) activates UPR^mt^, slowing muscle aging in elderly mice. Acipimox improves mitochondrial function in human skeletal muscle by activating UPR^mt^. Silencing miR-382-5p can also activate UPR^mt^ in skeletal muscle, enhancing mitochondrial metabolism. Exercise, including high-intensity training, aerobic exercise, and endurance training, activates UPR^mt^, significantly enhancing protein homeostasis and providing protective effects on skeletal muscle. The LST combination of LIN28A, TERT, and sh-p53 delays the senescence of adult muscle progenitor cells by activating UPR^mt^.

High-intensity exercise training [146] or aerobic exercise [147] can induce imbalances in mitochondrial nuclear division in aged mouse skeletal muscles, activating UPR^mt^, significantly improving protein homeostasis, and providing protective effects on skeletal muscles [148, 149]. Inhibiting UPR^mt^ activation in skeletal muscles leads to weight gain, reduced physical activity, and decreased aerobic capacity in mice [150]. Another study on hyperinsulinemic and hyperglycemic Slc2a4+/- mice showed that endurance training is effective in improving mitochondrial activity and UPR^mt^ in skeletal muscles, while strength exercise mainly affects post-translational mechanisms and protein synthesis in skeletal muscles [151]. Compared to ordinary individuals, strength athletes have increased mitochondrial cristae density, smaller mitochondria size, and increased surface-to-volume ratio in their mitochondrial pool. Besides mitochondrial fusion-fission proteins, UPR^mt^ activation might also be involved in altering the mitochondrial phenotype [135]. In clinical studies, athletes exhibited higher levels of HSP 60 protein compared to obese individuals and type 2 diabetes patients, potentially related to UPR^mt^ activation induced by mitochondrial nuclear proteins [152]. Conversely, a study on zebrafish indicated that regular exercise activates mitochondrial biogenesis but inhibits UPR^mt^, improving mitochondrial homeostasis [153]. Functions of UPR^mt^ in myocardium and skeletal muscle are summarized in Figure 4.

## 5. Limitation, Conclusion and prospects

The review is subject to certain limitations. Firstly, the limited number of studies on skeletal muscle aging and UPRmt makes it challenging to consolidate the relevant findings into a comprehensive whole. This scarcity also leads to discrepancies within the manuscript, as divergent study outcomes lack sufficient cross-validation. Secondly, our efforts to synthesize and summarize the articles are based on their commonalities, potentially overlooking other valuable information for more in-depth discussions.

In this review, we have summarized the regulatory mechanisms of UPR^mt^ in C. elegans and mammals. In C. elegans, the regulatory mechanism of UPR^mt^ is primarily centered around ATFS-1, with other molecules mainly regulating UPR^mt^ through ATFS-1, making this mechanism relatively simple and clear. However, the regulation of UPR^mt^ in mammals is considerably more complex. The homologous protein ATF5 may play a central role in this process, with a regulatory network composed of various molecular pathways jointly regulating the activation of UPR^mt^. Notably, UPR^mt^ activation is not limited to the same cell but exists as cell non-autonomous UPR^mt^ [39]. To our knowledge, cell non-autonomous UPR^mt^ has only been found in non-mammalian organisms like C. elegans and Drosophilas, with the intestine being the only identified target organ [41, 42, 102]. Many mechanisms of cell non-autonomous UPR^mt^ remain to be elucidated. For example, are there other target organs for cell non-autonomous UPR^mt^? Does cell non-autonomous UPR^mt^ exist in mammals? What are its regulatory mechanisms and functions?

Early studies found that activating UPR^mt^ in C. elegans can extend lifespan, making it an important molecular target for delaying aging [86]. Unfortunately, there is currently no direct evidence proving that activating UPR^mt^ can extend lifespan in mammals. Besides the extremely complex regulation mechanisms of UPR^mt^ in mammals making precise intervention difficult, long-term activation of UPR^mt^ might also bring adverse effects to the organism [26, 154]. Additionally, studies in C. elegans indicate that only activating UPR^mt^ during development can extend lifespan, whereas activation during adulthood has adverse effects [41, 42]. Therefore, in mammals or even humans, is it necessary to activate UPR^mt^ briefly during a specific period to effectively extend lifespan?

Due to the slow replication of cardiomyocytes, regulating cardiac hypertrophy plays an important role in cardiac aging. In the heart, activating UPR^mt^ can improve cardiac hypertrophy and delay cardiac aging [104]. Interestingly, there is a significant connection between UPR^mt^, mitophagy and apoptosis. We hypothesize that UPR^mt^, together with mitophagy and apoptosis, constitutes a series of stress responses to maintain cardiomyocyte homeostasis and function, ensuring cardiomyocyte survival and coping with cardiac aging. UPR^mt^ also plays an important role in regulating skeletal muscle aging. Activating UPR^mt^ can improve skeletal muscle aging and delay the onset of sarcopenia [72, 137]. This is also a key mechanism by which exercise improves skeletal muscle function and delays aging [148, 149]. Therefore, activating UPR^mt^ to improve sarcopenia progression might be a potentially important molecular target. In addition, despite numerous studies demonstrating the benefits of UPR^mt^, some research indicates potential harmful effects of UPR^mt^ on overall lifespan or in striated muscle [26,121, 153,154].

**Figure 5.**
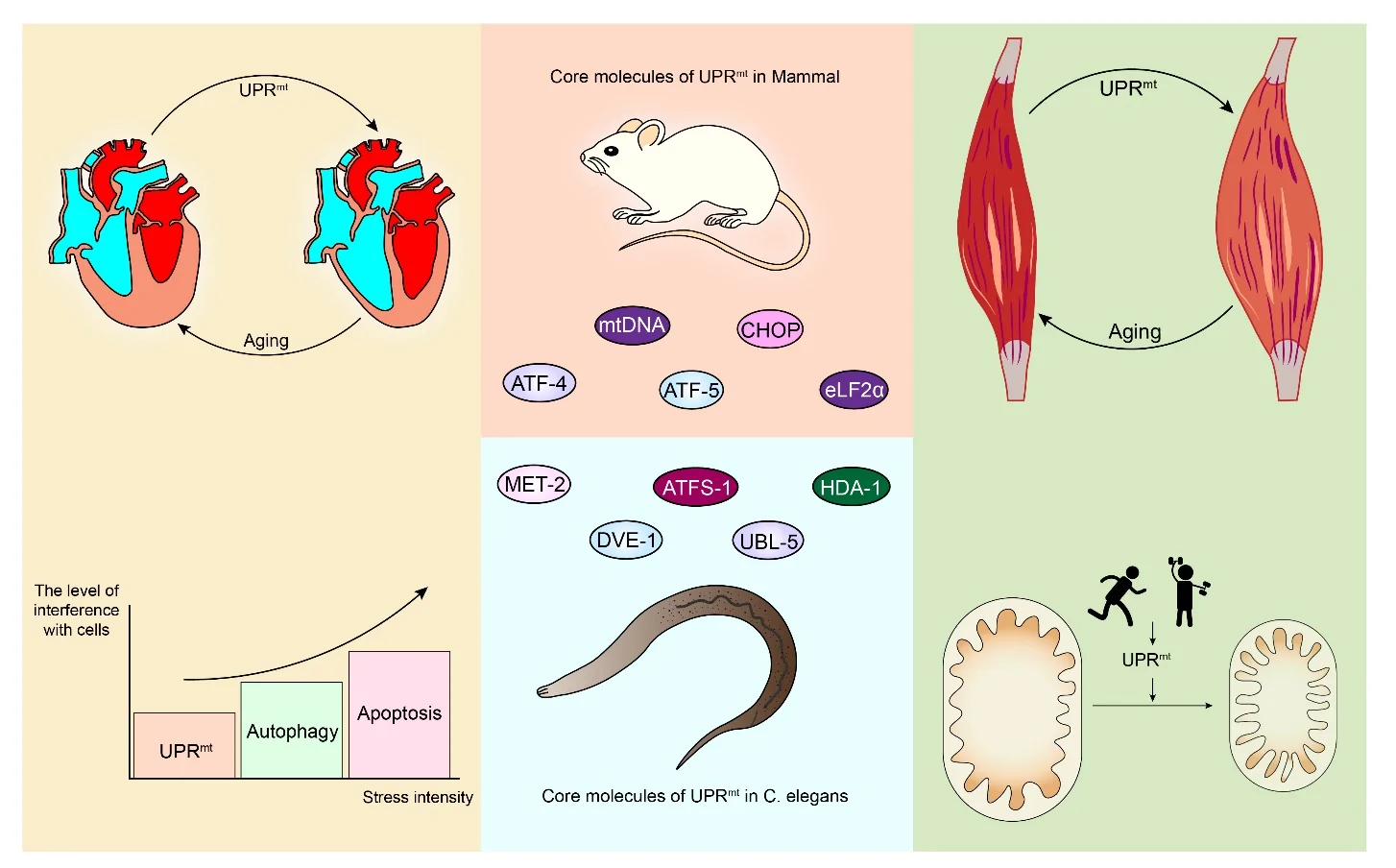
**The core molecules of UPR^mt^ in C**. elegans and mammals and the roles of UPRmt in cardiac and skeletal muscles.

The UPR^mt^ represents a conservative and extensive stress response system with multiple signaling pathways involved in both intracellular and intercellular communications, maintaining protein homeostasis within mitochondria and ensuring cell survival (Fig. 5). It plays a crucial role in various physiological functions, including lifespan extension and mitochondrial structural improvements. Given the growing global aging population, the associated socio-economic burden of striated muscle aging is increasing rapidly [155]. Further research is needed to explore how UPR^mt^ can delay striated muscle aging and mitigate the burden of age-related muscular diseases. Additionally, UPR^mt^ modulates molecular regulations in neurodegenerative disorders, diabetes, and cancer. Despite the relatively limited research on UPR^mt^, its significant potential for restoring protein homeostasis and enhancing mitochondrial function is conducive to the development of novel therapeutic strategies.
